# Association of Different Restriction Levels With COVID-19-Related Distress and Mental Health in Somatic Inpatients: A Secondary Analysis of Swiss General Hospital Data

**DOI:** 10.3389/fpsyt.2022.872116

**Published:** 2022-05-03

**Authors:** Nicola Julia Aebi, Günther Fink, Kaspar Wyss, Matthias Schwenkglenks, Iris Baenteli, Seraina Caviezel, Anja Studer, Sarah Trost, Sibil Tschudin, Rainer Schaefert, Gunther Meinlschmidt, the SomPsyNet Consortium

**Affiliations:** ^1^Swiss Tropical and Public Health Institute, Allschwil, Switzerland; ^2^University of Basel, Basel, Switzerland; ^3^Institute of Pharmaceutical Medicine (ECPM), University of Basel, Basel, Switzerland; ^4^Department of Psychosomatic Medicine, University Hospital and University of Basel, Basel, Switzerland; ^5^Department of Health Canton Basel-Stadt, Division of Prevention, Basel, Switzerland; ^6^Department of Geriatric Medicine FELIX PLATTER, University of Basel, Basel, Switzerland; ^7^Department of Obstetrics and Gynecology, University Hospital and University of Basel, Basel, Switzerland; ^8^Division of Clinical Psychology and Cognitive Behavioural Therapy, International Psychoanalytic University Berlin, Berlin, Germany; ^9^Division of Clinical Psychology and Epidemiology, Department of Psychology, University of Basel, Basel, Switzerland

**Keywords:** pandemic, depression, anxiety, health-related quality of life, social support

## Abstract

**Background:**

The coronavirus disease 2019 (COVID-19) pandemic and related countermeasures hinder health care access and affect mental wellbeing of non-COVID-19 patients. There is lack of evidence on distress and mental health of patients hospitalized due to other reasons than COVID-19—a vulnerable population group in two ways: First, given their risk for physical diseases, they are at increased risk for severe courses and death related to COVID-19. Second, they may struggle particularly with COVID-19 restrictions due to their dependence on social support. Therefore, we investigated the association of intensity of COVID-19 restrictions with levels of COVID-19-related distress, mental health (depression, anxiety, somatic symptom disorder, and mental quality of life), and perceived social support among Swiss general hospital non-COVID-19 inpatients.

**Methods:**

We analyzed distress of 873 hospital inpatients not admitted for COVID-19, recruited from internal medicine, gynecology, rheumatology, rehabilitation, acute geriatrics, and geriatric rehabilitation wards of three hospitals. We assessed distress due to the COVID-19 pandemic, and four indicators of mental health: depressive and anxiety symptom severity, psychological distress associated with somatic symptoms, and the mental component of health-related quality of life; additionally, we assessed social support. The data collection period was divided into modest (June 9 to October 18, 2020) and strong (October 19, 2020, to April 17, 2021) COVID-19 restrictions, based on the Oxford Stringency Index for Switzerland.

**Results:**

An additional 13% (95%-Confidence Interval 4–21%) and 9% (1–16%) of hospital inpatients reported distress related to leisure time and loneliness, respectively, during strong COVID-19 restrictions compared to times of modest restrictions. There was no evidence for changes in mental health or social support.

**Conclusions:**

Focusing on the vulnerable population of general hospital inpatients not admitted for COVID-19, our results suggest that tightening of COVID-19 restrictions in October 2020 was associated with increased COVID-19-related distress regarding leisure time and loneliness, with no evidence for a related decrease in mental health. If this association was causal, safe measures to increase social interaction (e.g., virtual encounters and outdoor activities) are highly warranted.

**Trial registration:**

www.ClinicalTrials.gov, identifier: NCT04269005.

## Introduction

Coronavirus disease 2019 (COVID-19) can interfere with health care delivery, and negatively affect mental health (1–3). Beyond SARS-CoV-2 infections, impeded health care for non-COVID-19 patients is a major threat (4). During the first wave of the COVID-19 pandemic, limited access to health care was reported (2, 5, 6). Despite the decrease of admissions to hospitals, admissions due to mental health issues raised in the United Kingdom (5). Therefore, elucidating distress and mental health of hospital inpatients not admitted for COVID-19 is of paramount importance. Poor mental health is further associated with chronic diseases (7). Thus, different calls for research on the vulnerable population of individuals with chronic diseases were published (8, 9). This research should also include hospital inpatients presenting with various somatic diseases, such as diseases related to internal medicine, gynecology, rheumatology, rehabilitation, geriatrics, and others. This population is specifically vulnerable for COVID-19 and severe courses including mortality (given the risk factors: physical disease and older age), and for not sufficiently seeking or receiving health care for non-COVID-19 related physical illness (which per definition all of them have). However, studies on distress and mental health of hospital inpatients not admitted for COVID-19 are missing, whereas evidence from studies focusing on populations with chronic diseases remains inconclusive, as was also found in a systematic review comparing the mental health impact of COVID-19 on vulnerable and non-vulnerable groups (10). While some studies suggest increased prevalence of depression, anxiety, and distress (11, 12), others report no indications for an association between mental health and the COVID-19 pandemic on people with pre-existing chronic disease (13, 14). However, most of the present literature is cross-sectional focusing on one point or period in time. The few longitudinal studies available to date report either small or no associations of COVID-19 restrictions with mental health outcomes in the general population (15, 16). Although evidence for the population of hospital inpatients not admitted for COVID-19 is missing, the inconsistent results regarding the association of COVID-19 restrictions with mental health may result from a combination of negative effects of the COVID-19 pandemic in some sub-groups, together with positive effects of the COVID-19 pandemic on quality of life and social support in other sub-groups. For instance, in countries such as the United Kingdom, the United States, and New Zealand people had the ability to save money due to lower consumption levels and lower risk of job loss (17), more flexibility at work, and less commuting (18), allowing for more time for personal growth, family and close friends (17–19).

Social networks are a protective factor against depression, anxiety, and other mental health problems (20). Social support from family and friends may have helped to prevent and address mental health symptoms, which occurred during the COVID-19 pandemic (21). Between June and October 2020, modest COVID-19 restrictions (the mean Oxford Stringency Index, which ranges from 0 to 100, was 39.1 in Switzerland) allowed maintaining social contacts in Switzerland. However, stronger COVID-19 restrictions (mean Oxford Stringency Index in Switzerland was 63.4) introduced in October 2020, such as home office and restrictions in leisure activities, may have impeded social contacts and likely, mental health. Lack of a social network may have impaired mental health, especially during quarantine and isolation (22, 23). Moreover, in the United States personal distancing was associated with more mental health symptoms, independent from stay-at-home orders (24). A Swiss study conducted at an emergency department reported fewer admissions due to suicidal behavior during lockdown as compared to after the lockdown (25). This finding was supported by a meta-analysis of longitudinal studies that did not find increased suicide rates during lockdown, which the authors explained by social cohesion (15), highlighting the importance of social contacts, especially among vulnerable groups. Taken together, social support seems to be an important protective factor for mental health, which may be compromised due to COVID-19 restrictions.

Therefore, our aim was to assess general hospital inpatients' COVID-19-related distress, mental health, and social support during periods of modest and strong COVID-19 restrictions defined by the Oxford Stringency Index. Based on the above mentioned finding that the COVID-19 pandemic was associated with poorer mental health outcomes in some populations and that COVID-19 restrictions may reduce social networks, we hypothesized that strong COVID-19 restrictions, as implemented in Switzerland from October 2020 to April 2021, were related to (i) increased COVID-19-related distress, (ii) poorer mental health outcomes, and (iii) less social support in general hospital inpatients not admitted for COVID-19.

## Methods

### Study Setting

We conducted this secondary analysis using prospective data collected as part of an ongoing clinical trial aiming at the early identification and management of elevated psychosocial distress among inpatients in three general hospitals in Basel, Switzerland. The SomPsyNet project includes patients from SOMatic hospitals with the objective to promote the prevention of PSYchological distress by establishing a stepped and collaborative care NETwork (NCT04269005) (26).

The Ethics Committee of Northwest and Central Switzerland approved the study protocol (2019–01724), including an amendment that contained COVID-19-related questions to assess the impact of the COVID-19 pandemic on psychosocial distress. All patients gave written informed consent.

### Study Population

Adult non-COVID-19 general hospital inpatients admitted for somatic health problems across nine hospital wards, including internal medicine, gynecology, rheumatology, rehabilitation, acute geriatrics, and geriatric rehabilitation, were eligible to participate in SomPsyNet. The following exclusion criteria applied: age below 18 years, not understanding/speaking German, not being able to give informed consent personally, not being able to follow the procedures of the study due to severe medical issues, risk of current suicidality or attempted suicide, and oncological conditions (due to existing standardized psycho-oncological care). Supplementary Figure 1 depicts a detailed flow-chart, emphasizing that most admitted hospital inpatients were either not eligible for SomPsyNet or refused to participate, reached the time limit, or left the hospital already.

### Study Design

We collected outcome data at baseline between June 9, 2020, and April 17, 2021. Within 72 h after admission to the hospital, study staff asked hospital inpatients not admitted for COVID-19 enrolled in the SomPsyNet study to complete a detailed questionnaire. We collected data using the platform “Heartbeat One” (provided by Heartbeat Medical Solutions GmbH, Berlin, Germany).

We used the Oxford Stringency Index (ranging from 0 to 100) for Switzerland, provided by the *Konjunkturforschungsstelle* (KOF; Swiss Economic Institute) (27) to divide the study into two periods. This index is based on nine indicators including school/workplace closing, cancellation of public events, restrictions on gatherings, closure of public transport, stay-at-home requirements, restriction on internal movement, international travel controls, and public information campaigns (27). We determined the time point when the Swiss government added again COVID-19 restrictions after a period with modest restrictions to distinguish between a period with modest and a period with strong COVID-19 restrictions, October 19, 2020. As illustrated in Table 1, the initial recruitment period (June 9 to October 18, 2020) was characterized by modest restrictions as the Swiss government had lifted most of the previous restrictions. Due to rising numbers of COVID-19 cases, the Swiss government imposed stronger restrictions again in October 2020, including restrictions on public gatherings and other leisure activities as well as closure of restaurants and non-essential stores. Also, there were restrictions in visitors' regulations at Swiss hospitals. These were hospital specific and varied in terms of timing and severity of implementation across the included hospitals.

**Table 1:** Overview of the coronavirus disease 2019 (COVID-19) restrictions in the study period from June 9, 2020, to April 17, 2021.

**Modest COVID-19 restrictions (June 9, 2020–October 18, 2020)**	**Strong COVID-19 restrictions (October 19, 2020–April 17, 2021)**
•Hygiene measures •Wearing masks •Quarantine after travels from countries with increased risk of infection •Prohibition of major events •Contact tracing	In addition to the ones of the previous period •Restrictions of social gatherings •Closure of restaurants, stores for non-everyday needs, and cultural venues (e.g., museums) •Home office obligation •Restrictions in leisure activities (e.g., prohibition of leisure activities with more than five people)

### Variables

The survey contained questions on sociodemographic factors, general mental distress measures, COVID-19-related distress, and social support.

#### Patient Characteristics

Patient characteristics included self-reported sex, age, nationality, marital status, education, and the somatic symptom severity assessed by the 8-item Somatic Symptom Scale (SSS-8). Age was grouped into <65 year-old hospital inpatients and those of ≥65 years. Nationality included Swiss, German, French, and Others, which consists of all other nationalities and hospital inpatients with more than one nationality. Marital status was split into single, married, widowed, divorced, and other. Education level was separated into primary level or less, secondary level I, secondary level II, tertiary level, and other. The SSS-8 is validated in German and is a reliable tool to assess the somatic symptom severity, consisting of a five-point Likert scale (0–4), ranging from 0 to 32 (28). To describe the sample, we categorized the hospital inpatients into a lower (score <16) and a higher (score ≥ 16) level of somatic symptom severity.

#### COVID-19-Related Distress

To determine specific distress related to the COVID-19 pandemic in different life areas, we asked hospital inpatients not admitted for COVID-19: “How distressed were you by the COVID-19 or corona pandemic in the past week regarding...: (a) your economic/financial situation, (b) your physical constraints, (c) your nutrition/weight, (d) alcohol/nicotine/other substances, (e) insecurities/worries/anxieties related to health or medical treatment, (f) your work/education/retirement, (g) your private environment including family/(grand-)children/childcare/living situation and others, (h) your leisure activities/restrictions of personal freedom or others, (i) your loneliness, and (j) your emotional problems, such as sadness, depression, anxiety.” We derived these life areas from the monitoring of the impact of the COVID-19 pandemic on the Swiss general population conducted by the research institute Sotomo (29). The hospital inpatients not admitted for COVID-19 stated whether due to the COVID-19 pandemic, they were “substantially less distressed”, “slightly less distressed”, “neither less nor more distressed”, “slightly more distressed”, or “substantially more distressed”. For this analysis, we created a binary indicator for distress severity of each life area combining the groups “substantially less distressed”, “slightly less distressed”, and “neither less nor more distressed” to indicate not distressed hospital inpatients, and the groups “slightly more distressed” and “substantially more distressed” to indicate distressed hospital inpatients.

#### Mental Health

We assessed mental health through several validated and reliable tools: depressive symptom severity with the 8-item Patient Health Questionnaire (PHQ-8), anxiety severity with the 7-item General Anxiety Disorder questionnaire (GAD-7), psychological distress associated with somatic symptoms with the 12-item Somatic Symptom Disorder questionnaire (SSD-12), and mental quality of life with the mental component summary scale (MCS) of the Short Form 36, version 1 (SF-36v1) (30–33). The SSD-12 consists of a five-point Likert scale (0–4), with a total score ranging from 0 to 48 (32). PHQ-8 and GAD-7 are composed of a four-point Likert scale (0–3), with total scores ranging from 0 to 24 and 0 to 21, respectively (30, 34). While higher scores in PHQ-8, GAD-7, and SSD-12 stand for worse mental health, a higher score in the MCS of the SF-36v1 represents better mental quality of life (30, 32, 34, 35). For this analysis, we created binary variables for each mental health assessment tool indicating whether the patient was distressed or not. Aligning to other studies, we defined the cutoff for being distressed as follows: PHQ-8 score ≥ 10, GAD-7 score ≥ 10, SSD-12 score ≥ 23, and SF-36 MCS score ≤ 38 (30, 31, 36, 37).

#### Social Support

To assess social support of hospital inpatients not admitted for COVID-19, we used the Oslo Social Support Scale (OSSS-3). Following Bøen et al., we calculated sum scores ranging from 3 to 14 and categorized hospital inpatients not admitted for COVID-19 into receiving poor (3–8), moderate (9–11), or strong (12–14) social support (38).

### Statistical Methods

The analysis was conducted using STATA/IC 15.1 including only hospital inpatients not admitted for COVID-19 with complete data. We considered *p*-values smaller than 0.05 to be statistically significant. We used heteroscedasticity-robust standard errors in all analyses in this study to allow for a non-normal residual distribution.

First, we compared the characteristics of the sample population recruited during modest COVID-19 restrictions (pre-period) and the sample recruited during strong COVID-19 restrictions (post-period).

Second, we graphed unadjusted average weekly percentage of hospital inpatients stating COVID-19-related distress and poor mental health over the full study period, comparing average levels during the modest (pre-period) and strong (post-period) COVID-19 restrictions.

Third, we formally tested the association of COVID-19-related distress, mental health, and social support between the two periods of modest and strong COVID-19 restrictions using multiple regression models. We stratified the linear regression model by sex and age group, and tested whether associations differed between sex and age groups. We conducted these analyses both, with binary and continuous outcomes, and estimated similar models for social support. All multiple regression models were adjusted for sex, age group, nationality, marital status, education level, weekly incidence of COVID-19 infections in the canton of Basel-Stadt, and the hospital the inpatients were admitted to.

Following Wagner et al. (39), we also estimated interrupted time series (ITS) regression models as a sensitivity analysis for our main mental health outcomes (Supplementary Tables 1, 2). The ITS model included a linear time trend, a post-term, and an interaction term between time and post-term. The post-term captured the average change over time (shift in intercept), while the interaction-term captured the change in trends.

## Results

**Figure 1:**
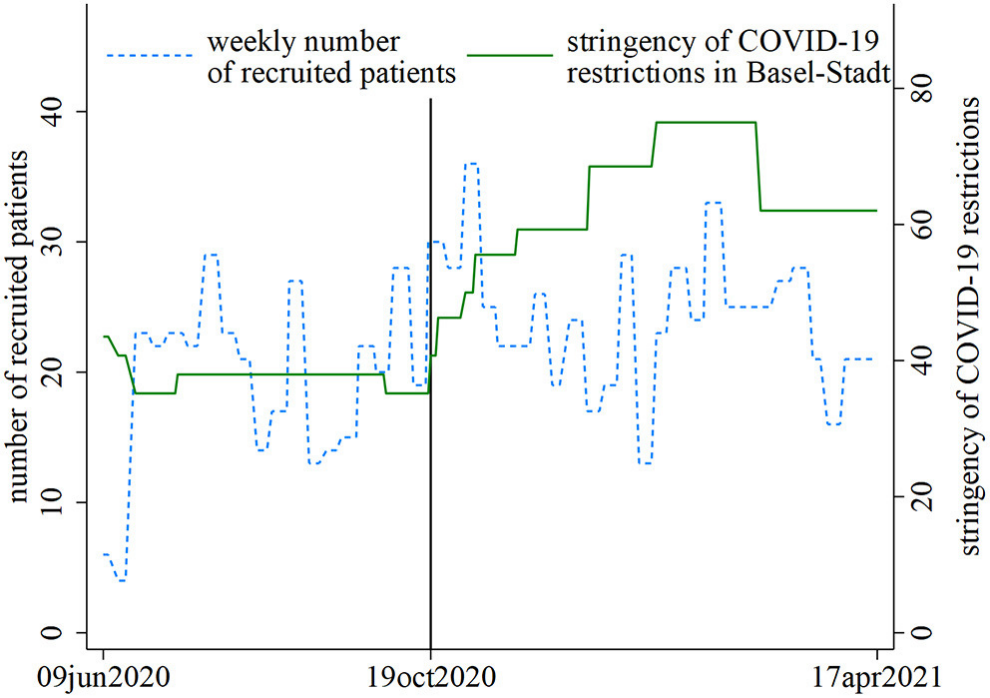
SomPsyNet recruitment (blue/dashed line) and stringency of coronavirus disease 2019 (COVID-19) restrictions in the canton of Basel-Stadt, Switzerland (green/solid line), in the study period. The black line separates the periods with modest (pre-period) vs. strong (post-period) COVID-19 restrictions.

Of 7,547 hospital inpatients admitted to the nine hospital wards, we included 873 hospital inpatients with complete data in this study (Supplementary Figure 1), whereby 324 hospital inpatients were recruited in the period of modest COVID-19 restrictions and 549 hospital inpatients in the period of strong COVID-19 restrictions. Figure 1 depicts the recruitment numbers (blue/dashed line) and the stringency of COVID-19 restrictions (green/solid line) in the study area during the two study periods before (pre-period: modest restrictions) and after (post-period: strong restrictions) the tightening of COVID-19 restrictions. The sociodemographic characteristics of hospital inpatients not admitted due to COVID-19 were similarly distributed in the two periods with modest and strong COVID-19 restrictions, except for admitting hospital, and medical field in which hospital inpatients were treated (Table 2). Another exception was sex: during modest COVID-19 restrictions, the proportion of females was lower than during strong COVID-19 restrictions, which is consistent with a marginally larger proportion of hospital inpatients not admitted for COVID-19 recruited at the level of the gynecology ward in the post-period period of strong COVID-19 restrictions.

**Table 2:** Patient characteristics, admitting hospital, and medical specialty of wards at which recruitment took place during modest (*n* = 324) and strong (*n* = 549) coronavirus disease 2019 (COVID-19) restrictions.

**Characteristics**	**Modest restrictions (pre-period)**		**Strong restrictions (post-period)**		***p*** -value*
	***n***	**%**	***n***	**%**	
Sex					
Male	154	47.5	218	39.7	
Female	170	52.5	331	60.3	0.024
Age group					
<65 years	177	54.6	303	55.2	
≥65 years	147	45.4	246	44.8	0.872
Nationality					
Swiss	235	72.5	425	77.4	
German	23	7.1	46	8.4	
French	2	0.6	5	0.9	
Other	64	19.8	73	13.3	0.082
Marital status					
Single	74	22.8	134	24.4	
Married	165	50.9	266	48.5	
Widowed	36	11.1	62	11.3	
Divorced	44	13.6	79	14.4	
Other	5	1.5	8	1.5	0.966
Highest education					
Primary level or less	11	3.4	21	3.8	
Secondary level I	53	16.4	68	12.4	
Secondary level II	141	43.5	235	42.8	
Tertiary level	108	33.3	215	39.2	
Other	11	3.4	10	1.8	0.170
Somatic Symptom Severity (SSS-8)					
Lower level (<16)	269	83.0	459	83.6	
Higher level (≥16)	55	17.0	90	16.4	0.823
Hospital					
University Hospital Basel	195	60.2	362	65.9	
University Department of Geriatric Medicine FELIX PLATTER	12	3.7	38	6.9	
Bethesda Hospital	117	36.1	149	27.1	0.006
Medical field					
Internal Medicine	165	50.9	270	49.3	
Gynecology	70	21.6	130	23.7	
Rheumatology	38	11.7	31	5.7	
Rehabilitation	39	12.0	79	14.4	
Acute geriatrics/geriatric rehabilitation	12	3.7	39	7.1	0.010

* *Comparison of modest and strong COVID-19 restrictions using Chi^2^-test*.

**Figure 2:**
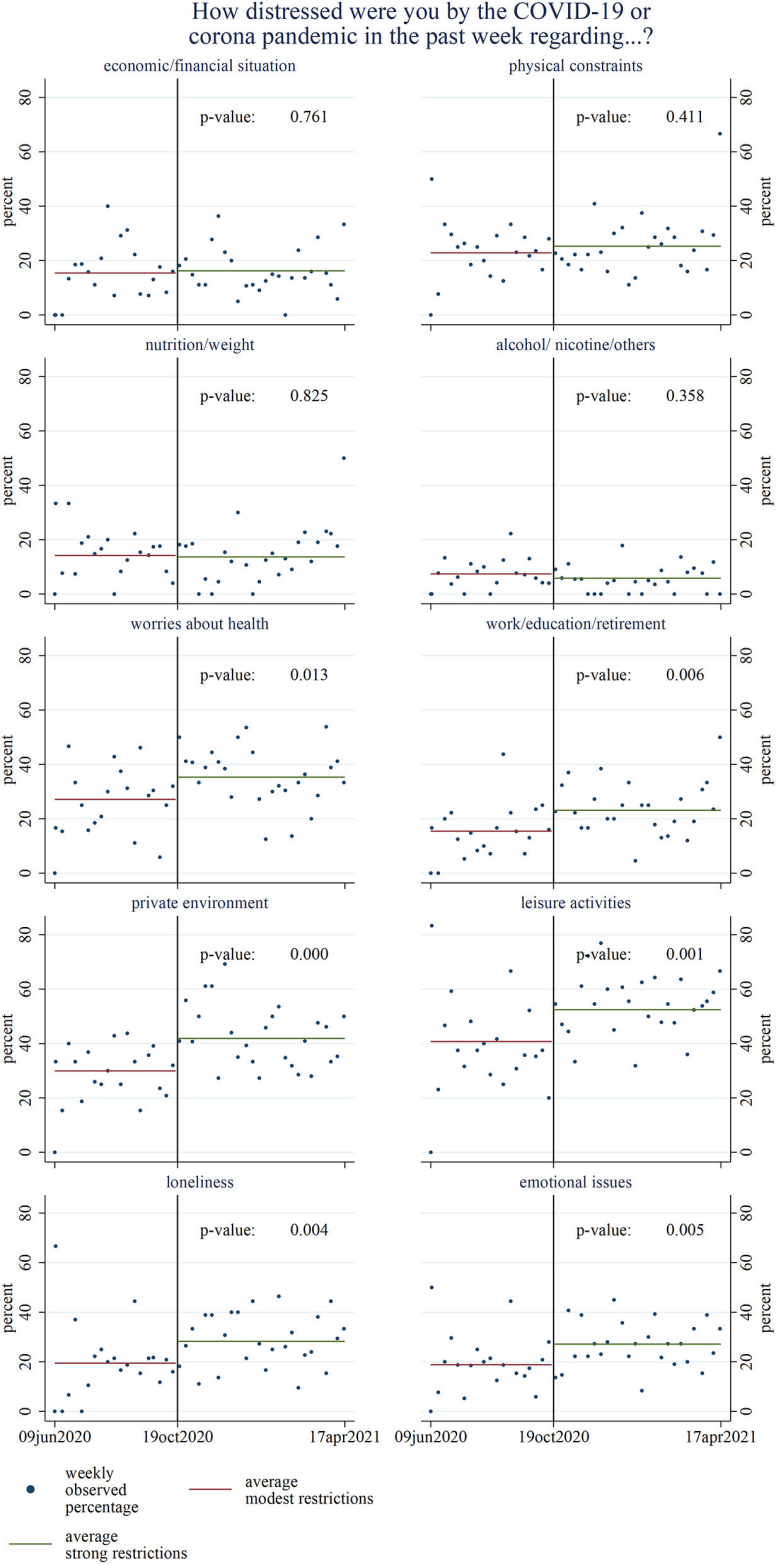
Comparison of weekly percentage of hospital inpatients stating being slightly or substantially more distressed due to the coronavirus disease 2019 (COVID-19) pandemic in the respective life area between the pre-period of modest and post-period of strong COVID-19 restrictions (*N* = 873). *P*-values are based on unadjusted linear regression analyses.

Unadjusted models showed that COVID-19-related distress increased significantly in six of ten life areas in the post-period of strong COVID-19 restrictions (Figure 2). No differences were found in distress regarding hospital inpatients' financial situation, physical constraints, nutrition, and alcohol, nicotine and similar substances intake. However, the percentage of hospital inpatients reporting more distress due to COVID-19 increased between 8% (95%-Confidence Interval [CI] 2–14%) in the life area of health or medical treatment and 12.0% (5.6–18.5%) in the area of private environment (including childcare and living situation) during strong as compared to modest restrictions. Continuous results did not indicate a change in distress scores regarding work/education/retirement and emotional problems, such as sadness, depression, anxiety, from modest to strong COVID-19 restrictions (Supplementary Figure 2).

Results differed after multivariable adjustment. When adjusting for sex, age group, nationality, marital status, education level, weekly incidence of COVID-19 infections in the canton of Basel-Stadt, and the hospital the inpatients were admitted to, the models (Table 3) only showed significant differences regarding leisure time, loneliness, and emotional issues, such as depression, sadness, or fears. Additionally, distress regarding physical complaints increased among older hospital inpatients not admitted for COVID-19 during the post-period of strong compared to the pre-period of modest COVID-19 restrictions. According to results from continuous data, only distress regarding leisure time increased significantly (Supplementary Table 3).

**Table 3:** Changes in the percentage of hospital inpatients reporting slightly or substantially more distress due to the coronavirus disease 2019 (COVID-19) pandemic in specific life areas and changes in perceived social support from periods of modest to strong COVID-19 restrictions, based on linear regression models, stratified by sex and age group (*N* = 873).

	**Change in percentage of hospital inpatients reporting increased distress during the period of strong restrictions (95%-CI)**				
	**All**	**Male**	**Female**	** <65 years**	**≥65 years**
Finances	−2.51 (−8.69 to 3.68)	−1.96 (−11.65 to 7.73)	−3.46 (−11.83 to 4.92)	−5.92 (−15.96 to 4.12)	2.77 (−4.52 to 10.06)
Physical complaints	4.46 (−3.10 to 12.02)	−3.00 (−15.14 to 9.14)	8.91 (−0.85 to 18.67)	−1.38 (−12.31 to 9.55)	11.72* (1.12 to 22.32)
Nutrition	2.28 (−4.04 to 8.59)	5.29 (−4.15 to 14.92)	−0.77 (−9.22 to 7.69)	−2.13 (−11.38 to 7.11)	7.80 (−0.93 to 16.53)
Alcohol, nicotine, others	−2.51 (−6.73 to 1.72)	−3.31 (−9.12 to 2.51)	−1.93 (−7.87 to 4.00)	−4.30 (−11.13 to 2.53)	0.16 (−4.27 to 4.59)
Worries about health	1.90 (−6.21 to 10.00)	−0.00 (−12.48 to 12.47)	3.36 (−7.54 to 14.27)	10.47 (−1.11 to 22.04)	−8.69 (−20.39 to 3.01)
Profession	4.41 (−2.21 to 11.04)	0.01 (−9.50 to 9.66)	6.90 (−1.90 to 15.71)	9.69 (−1.62 to 21.01)	−3.07 (−9.45 to 3.31)
Private environment	5.76 (−2.58 to 14.09)	5.49 (−7.23 to 18.21)	5.44 (−5.76 to 16.63)	8.83 (−3.02 to 20.68)	−1.28 (−12.97 to 10.41)
Leisure time	12.79** (4.09 to 21.48)	9.19 (−4.79 to 23.18)	14.68* (3.25 to 26.11)	10.08 (−1.95 to 22.11)	14.12* (1.10 to 27.13)
Loneliness	8.82* (1.27 to 16.38)	8.90 (−3.27 to 21.07)	8.74 (−1.23 to 18.71)	7.28 (−2.95 to 17.52)	11.40* (0.03 to 22.76)
Emotional issues	7.44 (−0.00 to 14.89)	2.01 (−9.13 to 13.15)	11.52* (1.24 to 21.79)	6.43 (−4.26 to 17.12)	9.10 (−1.34 to 19.54)
	**Change in mean score of social support** ^**§**^ **(95%-CI)**				
Social support (OSSS−3)	0.08 (−0.05 to 0.20)	0.21* (0.01 to 0.40)	−0.02 (−0.19 to 0.15)	0.08 (−0.10 to 0.26)	0.08 (−0.11 to 0.26)

*Results are adjusted for sex, age group, nationality, education level, marital status, weekly incidence of COVID-19 infections in Basel-Stadt, and hospital*.

* *p < 0.05*;

** *p ≤ 0.01*.

§ *Score from one (poor support) to three (strong support). CI, Confidence Interval; OSSS-3, Oslo Social Support Scale*.

Some sex and age differences regarding COVID-19-related distress were found (Supplementary Table 4). A higher proportion of females indicated increased COVID-19-related distress regarding physical complaints and emotional issues during strong compared to modest COVID-19 restrictions. Further, older hospital inpatients not admitted for COVID-19 reported less COVID-19-related distress due to health and profession during the post-period of strong compared to modest COVID-19 restrictions.

In total, 33.0% of general hospital inpatients not admitted for COVID-19 experienced strong social support while 19.1% stated to have poor social support. The mean social support did not significantly differ between the periods with modest and strong COVID-19 restrictions, except for males (Table 3). Males reported stronger social support during the period of strong COVID-19 restrictions compared to the pre-period with modest COVID-19 restrictions. However, this difference by sex regarding social support could not be confirmed in the equal coefficient analysis as depicted in Supplementary Table 4.

**Figure 3:**
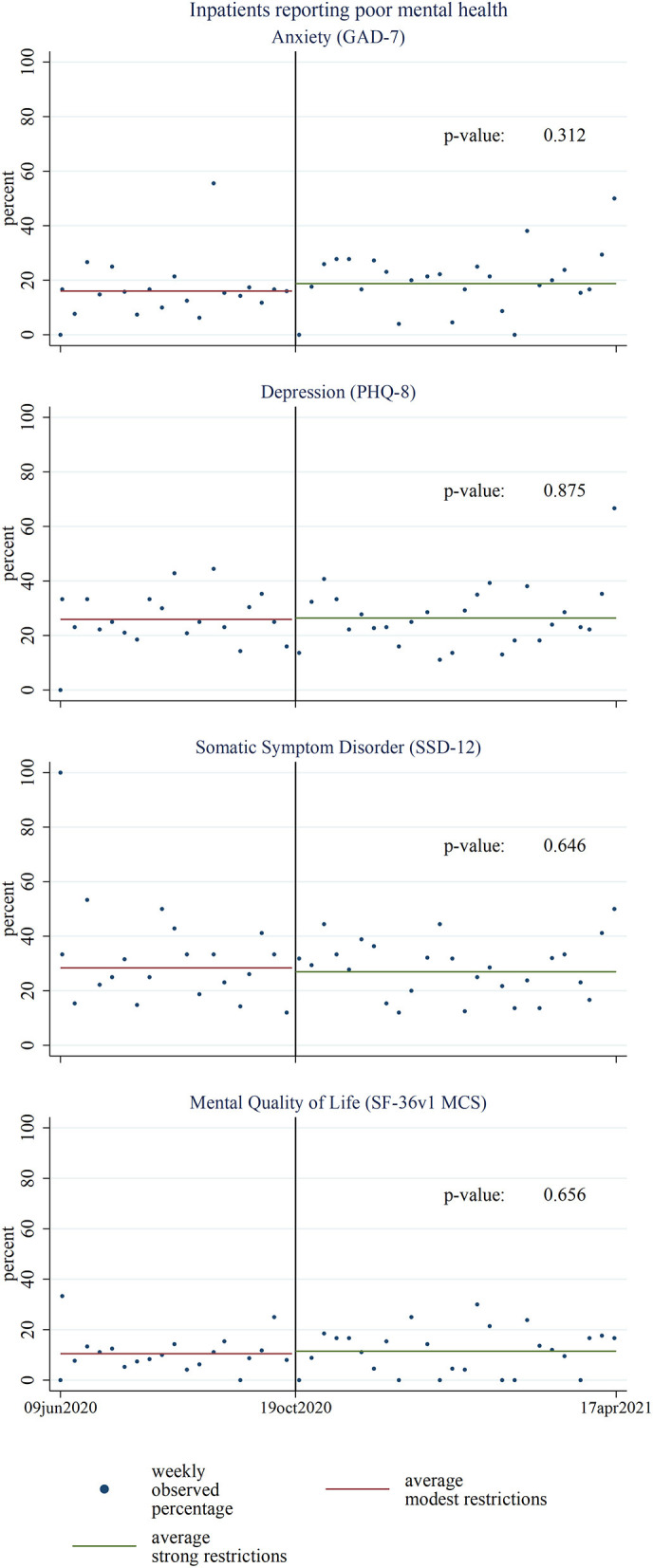
Comparison of percentage of hospital inpatients' mental health according to respective mental health assessment tools during the pre-period of modest and the post-period of strong coronavirus disease 2019 (COVID-19) restrictions (*N* = 873). *P*-values are based on unadjusted linear regression analyses. GAD-7, 7-item General Anxiety Disorder questionnaire; PHQ-8, 8-item Patient Health Questionnaire; SSD-12, 12-item Somatic Symptom Disorder questionnaire; SF-36v1, Short Form 36, version 1; MCS, mental component summary.

There was no evidence for differences in the percentage of hospital inpatients reporting poor mental health between the pre-period with modest to the post-period with strong COVID-19 restrictions in unadjusted (Figure 3) and adjusted (Table 4) models. Results were comparable for mental health scores (Supplementary Figure 3 and Supplementary Table 5) and highly consistent across all four mental health assessment tools with unadjusted mean scores of 16.8 (15.7–18.0), 6.6 (6.1–7.2), 5.3 (4.8–5.8), and 65.7 (63.4–68.0) for SSD-12, PHQ-8, GAD-7, and the MCS scale of SF-36v1, respectively, during modest COVID-19 restrictions.

**Table 4:** Change in percentage of hospital inpatients with poor mental health according to the mental health assessment tools from periods of modest to strong coronavirus disease 2019 (COVID-19) restrictions, based on linear regression models (*N* = 873).

	**Change in percentage of distressed hospital inpatients (95%-CI)**
Anxiety (GAD-7)	1.68 (−5.10 to 8.45)
Depression (PHQ-8)	−1.43 (−9.23 to 6.37)
Somatic Symptom Disorder (SSD-12)	−5.55 (−13.34 to 2.25)
Mental Quality of Life (SF-36v1 MCS)	1.81 (−3.82 to 7.43)

*Results are adjusted for sex, age group, nationality, education level, marital status, weekly incidence of COVID-19 infections in Basel-Stadt, and hospital. CI, Confidence Interval; AD-7, 7-item General Anxiety Disorder questionnaire; PHQ-8, 8-item Patient Health Questionnaire; SF-36v1, Short Form 36, version 1; MCS, mental component summary*.

## Discussion

To the best of our knowledge, there are no data on the impact of COVID-19 restrictions on general hospital inpatients not admitted for COVID-19. In this study, we investigated the association between the intensity of COVID-19 restrictions with levels of COVID-19-related distress, mental health, and perceived social support among inpatients not admitted for COVID-19 in Swiss general hospitals. Our main findings were that general hospital inpatients not admitted for COVID-19 reported higher COVID-19-related distress in some life areas in the period of stronger COVID-19 restrictions compared to the pre-period of modest COVID-19 restrictions: The percentage of hospital inpatients reporting more COVID-19-related distress regarding leisure activities and loneliness increased by 13 and 9%, respectively, when stronger COVID-19 restrictions were in place. However, this did not go along with indications of worse mental health regarding anxiety (GAD-7), depressive symptoms (PHQ-8), psychological distress associated with somatic symptoms (SSD-12), and mental quality of life (SF-36v1 MCS). Also, there was no indication for changes in perceived social support coinciding with stronger COVID-19 restrictions.

Our findings are in line with other studies that observed distress but no mental health consequences of strong COVID-19 restrictions in populations with selected somatic diseases. Yet, in contrast to our study, these studies did not include hospital inpatients not admitted for COVID-19. A study of young adults with congenital heart disease reported that COVID-19 restrictions were associated with loneliness and concerns about the respondents' health but not with depression or anxiety symptoms (40). This is mirrored in our results where, after the substantial new restrictions on leisure activities and social interactions imposed by the Swiss government from October 2020 onwards, more general hospital inpatients not admitted for COVID-19 stated distress regarding loneliness, independent of sex and age group. Despite this fact, the social support of general hospital inpatients not admitted for COVID-19 did not change with strong COVID-19 restrictions. Similarly, studies in Greece and in the Netherlands observed high levels of distress but no symptoms of depression or anxiety, or changes in mental quality of life in patients with chronic disease and dialysis patients, respectively (14, 41). Also in line with present findings is a German study investigating a cohort of patients with pre-existing mental disorders reporting an increase of psychosocial burden in patients in April/May 2020 before normalizing to pre-pandemic levels in November/December 2020 (42). At the same time, symptoms of mental disorders only changed minimally (42).

The literature highlights a substantial mental health burden of the pandemic. Thereby, Chiesa et al. described a positive association between COVID-19 restrictions and depression or anxiety in the general population (2), although no evidence for long-term effects of COVID-19 on mental health was found (43). However, evidence on COVID-19 restrictions and their impact on individuals with chronic diseases is scarce. Studies in Brazil that did not include hospital inpatients not admitted for COVID-19 suggest that people with chronic diseases had a higher likelihood of aggravated depression and anxiety symptoms than people without chronic diseases during the first wave of the COVID-19 pandemic compared to before the pandemic (11).

Several reasons may explain why we did not find indications of an association between strong COVID-19 restrictions and mental health in general hospital inpatients not admitted for COVID-19. First, most of the specific measures during the period with stronger COVID-19 restrictions did not directly affect patients who were hospitalized. Second, during their stay at a general hospital, the included hospital inpatients presumably were primarily focused on their physical wellbeing and health, while COVID-19 and its restrictions may have temporarily faded into the background. This may have positively influenced patients' self-reported mental health. Third, hospital inpatients may have developed coping strategies, such as seeking emotional support or avoiding the stressor (e.g., reducing the consumption of COVID-19 news), which reduce negative impacts of distress on mental health (44, 45). Fourth, individual and societal resilience may have prevented an increase of mental health consequences resulting from strong restrictions, as already described by others (15, 46). Fifth, over time, the population may have adapted to the COVID-19 restrictions. Various studies observed a decrease in depressive symptoms and anxiety after an initial rise at the beginning of the COVID-19 pandemic (47–49).

### Strengths and Limitations

Our study has several strengths. First, the focus on general hospital inpatients not admitted for COVID-19 with different somatic complaints provides a relevant addition to the current knowledge because chronic illness is a risk factor for distress (7). Second, this study applied data collection over time as well as comprehensive measures on COVID-19-related distress, mental health, and social support. Third, this study included data covering a period in 2020 with modest COVID-19 restrictions and the second/third wave of the COVID-19 pandemic with stronger restrictions in Switzerland. Many other studies still refer to mental health during the first wave in 2020, which may be different from the effects of COVID-19 restrictions after multiple lockdowns (15). As a result, our work can contribute to the understanding of the effect of different levels of COVID-19 restrictions on mental health after exposure to the COVID-19 pandemic becoming routine.

Some limitations should be mentioned. First, the data do not include individual follow-up data. This would have allowed estimating changes in mental health within individual hospital inpatients between periods of modest and strong COVID-19 restrictions. Through the nature of our data, however, we were able to assess trends of COVID-19-related distress and mental health before and after a switch from modest to strong restrictions. Second, mental health consequences were assessed using self-reported data. Self-reporting tools, however, often overestimate mental health consequences compared to clinical interviews (50). As we were interested in the change of mental health from modest to strong COVID-19 restrictions, the used mental health assessment tools were sufficient. Third, our results cannot be transferred to the general population or to all hospital inpatients due to the restricted range of wards in which patients were recruited. Therefore, generalizability of results beyond patient groups from the specialties covered should be conducted with caution. Forth, observational data are prone to confounding. To account for this, we adjusted the statistical analysis for these factors. Fifth, smaller effects are of course possible, but could not be detected with the sample size we had. It is also possible that anxiety and depression do not respond immediately to short-term variations in external disease risk or government measures. Long-term follow-up studies would be required to answer this empirically. Sixth, it is possible that the differences in exposures were too small to see differential mental health outcomes. All measures were taken during the pandemic, just at different stages.

### Policy Implications and Future Research

The increased distress regarding leisure time and loneliness during strong COVID-19 restrictions indicates that promotion of alternative social interactions (e.g., virtual) and outdoor activities (e.g., walking) may be of great value to diminish distress levels. However, social support did not change with stronger COVID-19 restrictions in hospital inpatients not admitted for COVID-19. The present social support may have strengthened the individual resilience, and hence, alleviated detrimental mental health consequences in this vulnerable population. Future research should focus on pathways explaining why COVID-19-related distress does not result in mental health consequences. Specific aspects of interest are the individual and societal resilience in the context of changing COVID-19 restrictions, as well as potential temporal delays of mental health consequences. Qualitative research may add value to these aspects and may help to explain our results.

## Conclusion

Our results indicate that the pronounced tightening of COVID-19 restrictions in Switzerland, in the period October 2020 to April 2021, went along with higher COVID-19-related distress among general hospital inpatients not admitted for COVID-19 in Switzerland but did not associate with measurable changes in overall mental health. More specifically, hospital inpatients not admitted for COVID-19 felt more distressed regarding restrictions in leisure time and loneliness during times of strong COVID-19 restrictions. Therefore, social interactions (e.g., virtual) should be promoted to mitigate distress levels. More research is needed to understand the differing results regarding COVID-19-related distress and mental health.

## Data Availability Statement

The datasets presented in this article are not readily available as they are being held by the SomPsyNet project. In the case of inquiries by third parties that wish to reuse data after an embargo period, they may submit a project synopsis addressed to the Publications Committee of the SomPsyNet project and will have to obtain authorization of the responsible ethics committee as ordained in the Ordinance of 20 September 2013 on Human Research with the exception of Clinical Trials (Human Research Ordinance, HRO). The Publication Committee will review the project synopsis and will answer formal request of applicants. Only upon collection of all important consents and upon approval of the responsible Ethics Committee(s), the requested data will be transferred to the applicants. Third parties have to confirm and provide evidence to comply with all relevant Swiss and cantonal laws and regulations (especially regarding data protection and Human Research), as well as all obligations and regulations set out in the documents and contracts related to SomPsyNet. Fees may apply to cover expenses related to the data reuse. Requests to access the datasets should be directed to GM, gunther.meinlschmidt@unibas.ch.

## Ethics Statement

The studies involving human participants were reviewed and approved by Ethics Committee Northwest and Central Switzerland. The patients/participants provided their written informed consent to participate in this study.

## SomPsyNet Consortium

Johannes Beck (Clinic Sonnenhalde, Riehen, Switzerland), Christian G. Huber (Department of Psychiatry and Psychotherapy, University Psychiatric Clinics, University of Basel, Basel, Switzerland), Johanna Fremmer (Department of Psychosomatic Medicine, University Hospital and University of Basel, Basel, Switzerland), Florian F. Grossmann (Division of Nursing, Department of Medicine, University Hospital Basel, Basel, Switzerland), Maria C. Katapodi (Department of Clinical Research, University of Basel, Basel, Switzerland; University of Michigan School of Nursing, Ann Arbor, MI, United States), Robert C. Keller (Swiss Heart Foundation, Bern, Switzerland), Undine E. Lang (Department of Psychiatry and Psychotherapy, University Psychiatric Clinics, University of Basel, Basel, Switzerland), Lisa Schiess (Department of Psychosomatic Medicine, University Hospital and University of Basel, Basel, Switzerland), Nadine Schur (Institute of Pharmaceutical Medicine (ECPM), University of Basel, Basel, Switzerland), Sonja Seelmann (Department of Internal Medicine, University Hospital Basel, Basel, Switzerland), Thomas Steffen (Department of Health Canton Basel-Stadt, Medical Services, Basel, Switzerland).

## Author Contributions

NA, GF, KW, MS, IB, SC, AS, RS, and GM contributed to the design and conduct of the study. NA conducted the statistical analysis, wrote a first draft, and revised the manuscript. All authors contributed to the interpretation of the data and critically reviewed and revised the manuscript for important intellectual content. All authors read and approved the final manuscript and agreed to submit it for publication.

## Funding

The project SomPsyNet received funding from Health Promotion Switzerland (GFCH) under project no. PGV01_087 and was supported by intramural funds from the Department of Health, Canton of Basel-Stadt, and the Department of Psychosomatic Medicine, University Hospital and University of Basel. The external funding sources had no involvement in study design; in the collection, analysis, and interpretation of the data; in the writing of the report; and in the decision to submit the article/contribution for publication. Additional funds based on a performance agreement were provided by the Swiss Learning Health System.

## Conflict of Interest

The authors declare that the research was conducted in the absence of any commercial or financial relationships that could be construed as a potential conflict of interest.

## Publisher's Note

All claims expressed in this article are solely those of the authors and do not necessarily represent those of their affiliated organizations, or those of the publisher, the editors and the reviewers. Any product that may be evaluated in this article, or claim that may be made by its manufacturer, is not guaranteed or endorsed by the publisher.

## Abbreviations

CI: Confidence Interval
COVID-19: coronavirus disease 2019
EKNZ: Ethikkommission Nordwest- und Zentralschweiz Ethics Committee of Northwest and Central Switzerland
GAD-7: 7-item Generalized Anxiety Disorder
ITS: Interrupted Time Series
KOF: Konjunkturforschungsstelle (Swiss Economic Institute)
MCS: mental component summary scale
OSSS-3: Oslo Social Support Scale
PHQ-8: 8-item Patient Health Questionnaire
SF-36v1, Short Form 36: version 1
SSD-12: 12-item Somatic Symptom Disorder
SomPsyNet, comprehensive health care project for patients from SOMatic hospitals that promotes the prevention of PSYchosocial distress by establishing a stepped and collaborative care NETwork in Basel: Switzerland
SSS-8: 8-item Somatic Symptom Severity.
