# Supplementary material for: Association of Different Restriction Levels With COVID-19-Related Distress and Mental Health in Somatic Inpatients: A Secondary Analysis of Swiss General Hospital Data
Source: Front Psychiatry. 2022 May 3;13:872116. doi: 10.3389/fpsyt.2022.872116 (PMC9113023; doi:10.3389/fpsyt.2022.872116)
Supplement: Supplementary file 2 [file Image_2.pdf]

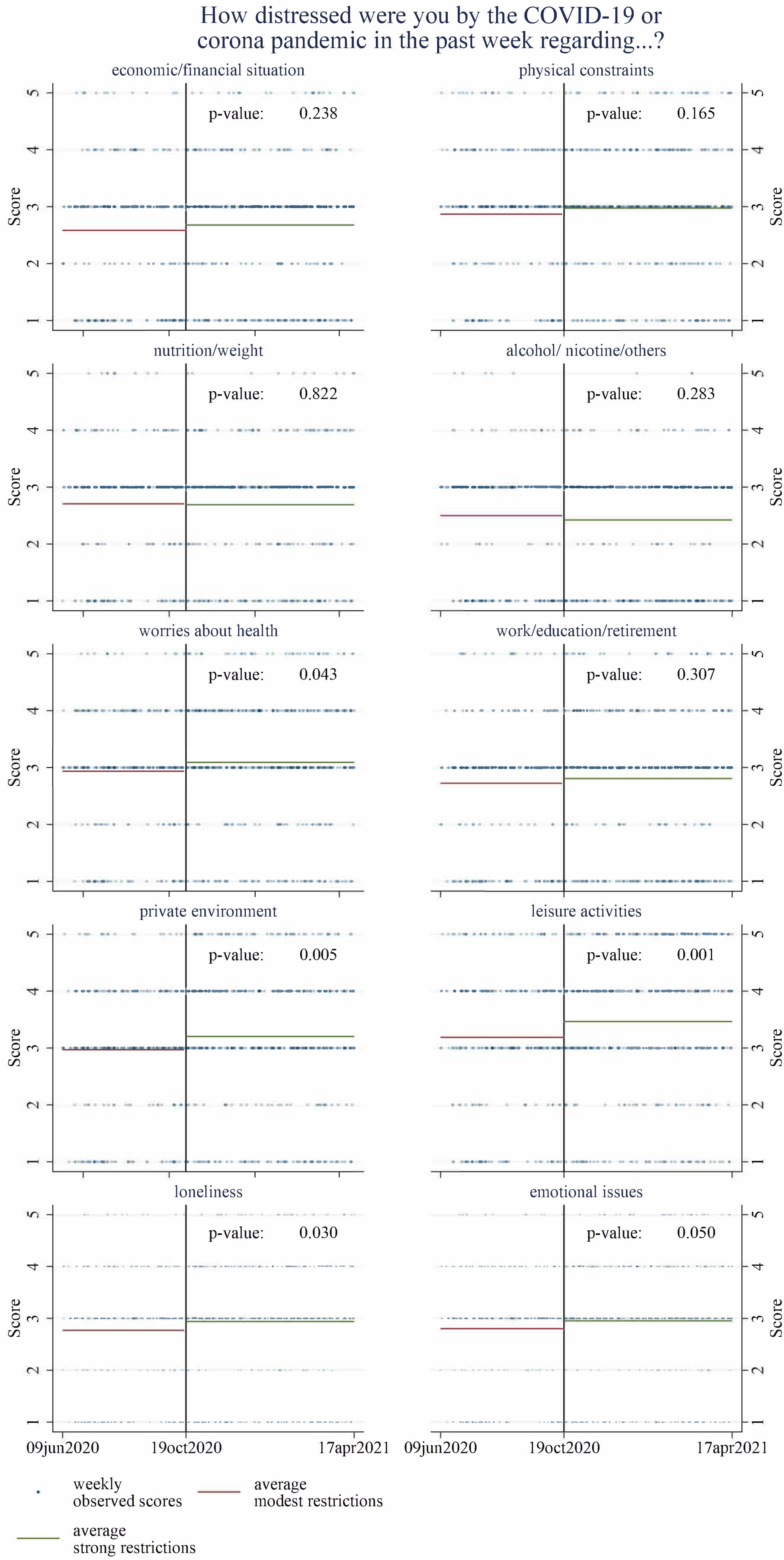

Supplementary Figure 2. Stated distress scores of inpatients due to coronavirus disease 2019 (COVID-19) pandemic in the respective life area between the pre-period of modest and the post-period of strong COVID-19 restrictions (*N* = 873). P-values are based on unadjusted linear regression analyses.
