# Supplementary material for: Association of Different Restriction Levels With COVID-19-Related Distress and Mental Health in Somatic Inpatients: A Secondary Analysis of Swiss General Hospital Data
Source: Front Psychiatry. 2022 May 3;13:872116. doi: 10.3389/fpsyt.2022.872116 (PMC9113023; doi:10.3389/fpsyt.2022.872116)
Supplement: Supplementary file 3 [file Image_3.pdf]

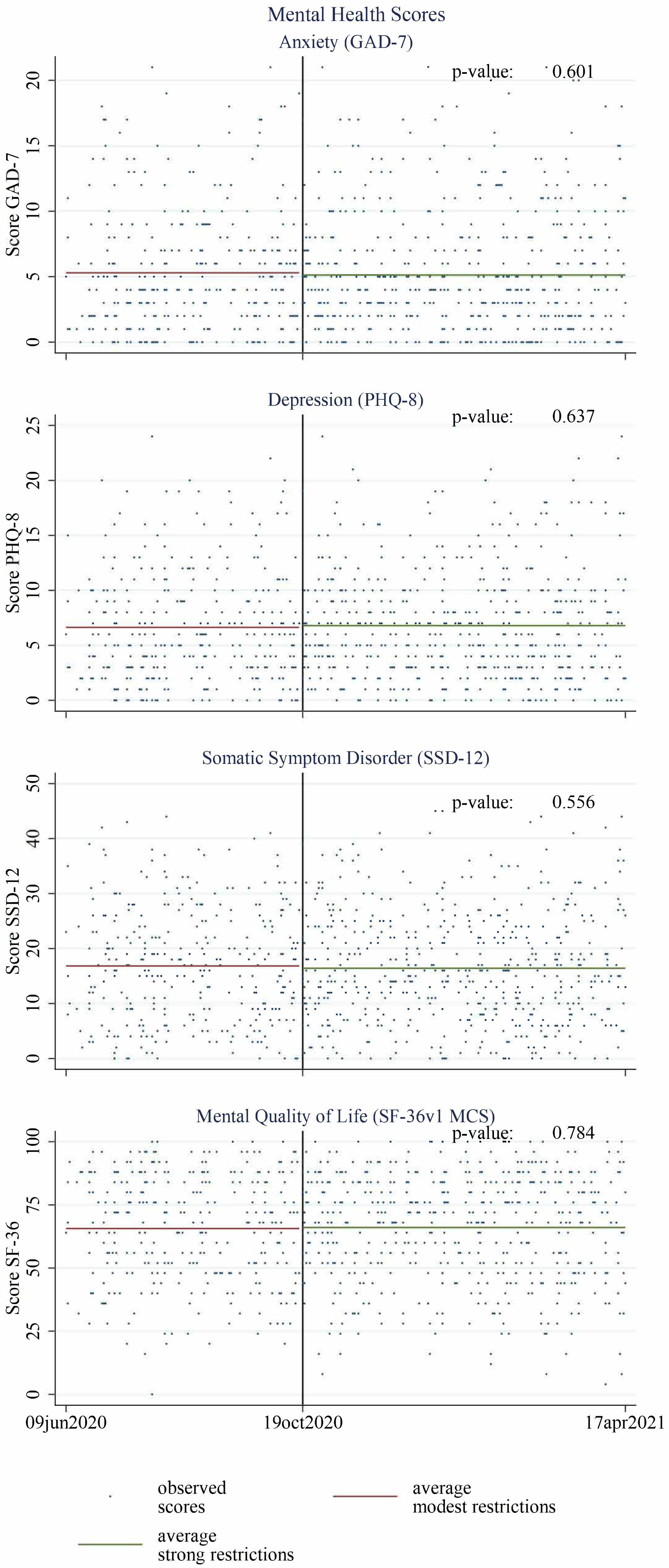

Supplementary Figure 3. Comparison of distress scores between the pre-period of modest and the post-period of strong coronavirus disease 2019 (COVID-19) restrictions (*N* = 873). P-values are based on unadjusted linear regressions.

GAD-7 = 7-item General Anxiety Disorder questionnaire
PHQ-8 = 8-item Patient Health Questionnaire
SSD-12 = 12-item Somatic Symptom Disorder questionnaire
SF-36v1 = Short Form 36, version 1
