# Supplementary material for: Association of Different Restriction Levels With COVID-19-Related Distress and Mental Health in Somatic Inpatients: A Secondary Analysis of Swiss General Hospital Data
Source: Front Psychiatry. 2022 May 3;13:872116. doi: 10.3389/fpsyt.2022.872116 (PMC9113023; doi:10.3389/fpsyt.2022.872116)
Supplement: Supplementary file 4 [file Table_1.docx]

| **Supplementary Table 1** Interrupted Time Series Regression Analyses of percentage of distressed inpatients according to the mental health assessment tools (*N* = 873). | | | |
| --- | --- | --- | --- |
|  | | Coefficient (95%-CI) | p-value |
| Anxiety (GAD-7) | |  |  |
| Time trend | | 0.01 (-0.11 to 0.12) | 0.903 |
| Change in mean (level) | | -18.25 (-46.58 to 10.07) | 0.206 |
| Change in time trend (slope) | | 0.06 (-0.09 to 0.22) | 0.393 |
| Depression (PHQ-8) | |  |  |
| Time trend | | -0.01 (-0.15 to 0.12) | 0.849 |
| Change in mean (level) | | -17.46 (-49.83 to 14.91) | 0.290 |
| Change in time trend (slope) | | 0.06 (-0.11 to 0.25) | 0.460 |
| Somatic Symptom Disorder (SSD-12) |  | |  |
| Time trend | | -0.02 (-0.13 to 0.23) | 0.745 |
| Change in mean (level) | | -14.44 (-47.51 to 18.64) | 0.392 |
| Change in time trend (slope) | | 0.05 (-0.13 to 0.23) | 0.600 |
| Mental Quality of Life (SF-36v1 MCS) | |  |  |
| Time trend | | 0.03 (-0.08 to 0.13) | 0.605 |
| Change in mean (level) | | -3.34 (-26.21 to 19.52) | 0.774 |
| Change in time trend (slope) | | -0.00 (-0.14 to 0.13) | 0.981 |
| Results are adjusted for sex, age group, nationality, education level, marital status, weekly incidence of COVID-19 infections in Basel-Stadt, and hospital.  CI = Confidence Interval GAD-7 = 7-item General Anxiety Disorder questionnaire PHQ-8 = 8-item Patient Health Questionnaire SSD-12 = 12-item Somatic Symptom Disorder questionnaire SF-36v1 = Short Form 36, version 1 MCS = mental component summary | | | |
