# Supplementary material for: Association of Different Restriction Levels With COVID-19-Related Distress and Mental Health in Somatic Inpatients: A Secondary Analysis of Swiss General Hospital Data
Source: Front Psychiatry. 2022 May 3;13:872116. doi: 10.3389/fpsyt.2022.872116 (PMC9113023; doi:10.3389/fpsyt.2022.872116)
Supplement: Supplementary file 5 [file Table_2.docx]

| **Supplementary Table 2** Interrupted Time Series Regression Analyses of distress scores (*N* = 873). | | | |
| --- | --- | --- | --- |
|  | | Coefficient (95%-CI) | p-value |
| Anxiety (GAD-7) | |  |  |
| Time trend | | 0.01 (-0.00 to 0.02) | 0.186 |
| Change in mean (level) | | 0.10 (-3.40 to 3.59) | 0.956 |
| Change in time trend (slope) | | -0.01 (-0.03 to 0.01) | 0.382 |
| Depression (PHQ-8) | |  |  |
| Time trend | | 0.01 (-0.01 to 0.02) | 0.386 |
| Change in mean (level) | | -0.71 (-4.38 to 2.97) | 0.706 |
| Change in time trend (slope) | | -0.00 (-0.02 to 0.02) | 0.812 |
| Somatic Symptom Disorder (SSD-12) |  | |  |
| Time trend | | -0.00 (-0.03 to 0.03) | 0.824 |
| Change in mean (level) | | -5.06 (-12.52 to 2.41 | 0.184 |
| Change in time trend (slope) | | 0.02 (-0.02 to 0.06) | 0.417 |
| Mental Quality of Life (SF-36v1 MCS^*^) | |  |  |
| Time trend | | -0.03 (-0.10 to 0.03) | 0.316 |
| Change in mean (level) | | -2.10 (-17.23 to 13.03) | 0.786 |
| Change in time trend (slope) | | 0.03 (-0.05 to 0.12) | 0.453 |
| Results are adjusted for sex, age group, nationality, education level, marital status, weekly incidence of COVID-19 infections in Basel-Stadt, and hospital. * A higher score indicates better mental health.  CI = Confidence Interval GAD-7 = 7-item General Anxiety Disorder questionnaire PHQ-8 = 8-item Patient Health Questionnaire SSD-12 = 12-item Somatic Symptom Disorder questionnaire SF-36v1 = Short Form 36, version 1 MCS = mental component summary | | | |
