# Supplementary material for: Association of Different Restriction Levels With COVID-19-Related Distress and Mental Health in Somatic Inpatients: A Secondary Analysis of Swiss General Hospital Data
Source: Front Psychiatry. 2022 May 3;13:872116. doi: 10.3389/fpsyt.2022.872116 (PMC9113023; doi:10.3389/fpsyt.2022.872116)
Supplement: Supplementary file 6 [file Table_3.docx]

| **Supplementary Table 3** Changes in the mean score of reported distress (one to five) due to the coronavirus disease 2019 (COVID-19) pandemic in specific life areas from periods of modest to strong COVID-19 restrictions based on linear regression models, stratified by sex and age group (*N* = 873). | | | | | | | | | |
| --- | --- | --- | --- | --- | --- | --- | --- | --- | --- |
|  | Mean change of COVID-19-related distress score (95%-CI) | | | | | | | | |
|  | All |  | Male |  | Female |  | <65 years |  | ≥65 years |
|  |  |  |  |  |  |  |  |  |  |
| Finances | 0.05 (-0.15 to 0.25) |  | 0.23 (-0.09 to 0.55) |  | -0.09 (-0.37 to 0.18) |  | -0.04 (-0.33 to 0.25) |  | 0.17 (-0.13 to 0.46) |
| Physical complaints | 0.13 (-0.06 to 0.33) |  | 0.17 (-0.16 to 0.49) |  | 0.09 (-0.16 to 0.34) |  | -0.07 (-0.33 to 0.19) |  | 0.34* (0.04 to 0.63) |
| Nutrition | 0.01 (-0.17 to 0.20) |  | 0.17 (-0.12 to 0.46) |  | -0.11 (-0.37 to 0.14) |  | -0.10 (-0.37 to 0.16) |  | 0.12 (-0.15 to 0.40) |
| Alcohol, nicotine, others | -0.13 (-0.32 to 0.05) |  | -0.02 (-0.30 to 0.27) |  | -0.19 (-0.44 to 0.06) |  | -0.17 (-0.42 to 0.08) |  | -0.11 (-0.38 to 0.17) |
| Worries about health | 0.06 (-0.13 to 0.26) |  | 0.16 (-0.16 to 0.48) |  | 0.01 (-0.25 to 0.27) |  | 0.12 (-0.15 to 0.38) |  | -0.03 (-0.33 to 0.27) |
| Profession | -0.01 (-0.21 to 0.19) |  | 0.02 (-0.30 to 0.33) |  | -0.02 (-0.29 to 0.25) |  | 0.01 (-0.28 to 0.29) |  | -0.08 (-0.37 to 0.21) |
| Private environment | 0.08 (-0.12 to 0.29) |  | 0.13 (-0.20 to 0.46) |  | 0.04 (-0.23 to 0.31) |  | 0.01 (-0.26 to 0.28) |  | 0.11 (-0.21 to 0.43) |
| Leisure time | 0.32** (0.11 to 0.54) |  | 0.32 (-0.04 to 0.68) |  | 0.33* (0.04 to 0.61) |  | 0.29 (-0.00 to 0.59) |  | 0.35* (0.02 to 0.67) |
| Loneliness | 0.17 (-0.02 to 0.37) |  | 0.29 (-0.03 to 0.62) |  | 0.12 (-0.15 to 0.38) |  | 0.05 (-0.23 to 0.32) |  | 0.32* (0.03 to 0.62) |
| Emotional issues | 0.16 (-0.04 to 0.35) |  | 0.23 (-0.10 to 0.55) |  | 0.14 (-0.11 to 0.39) |  | 0.06 (-0.21 to 0.33) |  | 0.25 (-0.04 to 0.54) |
| Results are adjusted for sex, age group, nationality, education level, marital status, weekly incidence of COVID-19 infections in Basel-Stadt, and hospital.  * p-value < 0.05; **p-value ≤ 0.01; *** p-value ≤ 0.001  CI = Confidence Interval | | | | | | | | | |
