# Supplementary material for: Association of Different Restriction Levels With COVID-19-Related Distress and Mental Health in Somatic Inpatients: A Secondary Analysis of Swiss General Hospital Data
Source: Front Psychiatry. 2022 May 3;13:872116. doi: 10.3389/fpsyt.2022.872116 (PMC9113023; doi:10.3389/fpsyt.2022.872116)
Supplement: Supplementary file 7 [file Table_4.docx]

| **Supplementary Table 4** Sex and age differences in changes in the percentage of inpatients reporting slightly or substantially more distress due to the coronavirus disease 2019 (COVID-19) pandemic in specific life areas, and changes in social support score from periods of modest to strong COVID-19 restrictions based on equal coefficient analyses (*N* = 873). | | | |
| --- | --- | --- | --- |
|  | Percentage change (95%-CI) | | |
|  | Sex (reference male) |  | Age (reference <65 years) |
| Finances | 0.67 (-9.43 to 10.77) |  | 4.10 (-5.58 to 13.79) |
| Physical complaints | 12.48* (0.61 to 24.35) |  | 4.68 (-7.05 to 16.42) |
| Nutrition | -3.24 (-12.77 to 6.30) |  | 4.14 (-5.26 to 13.55) |
| Alcohol, nicotine, others | 1.98 (-5.03 to 9.00) |  | 0.79 (-5.79 to 7.37) |
| Worries about health | 9.58 (-3.07 to 22.23) |  | -12.70* (-25.37 to -0.03) |
| Profession | 8.33 (-1.75 to 18.41) |  | -14.47** (-24.40 to -4.54) |
| Private environment | 6.65 (-6.39 to 19.69) |  | -4.93 (-17.87 to 8.00) |
| Leisure time | 3.32 (-10.43 to 17.06) |  | 3.43 (-10.22 to 17.08) |
| Loneliness | 4.80 (-6.86 to 16.45) |  | 2.19 (-9.41 to 13.79) |
| Emotional issues | 11.67* (0.37 to 22.97) |  | -0.61 (-11.93 to 10.70) |
|  |  |  |  |
|  | Change in mean social support score^§^ (95%-CI) | | |
| Social support (OSSS-3) | 0.00 (-0.00 to 0.00) |  | 0.00 (-0.00 to 0.00) |
| Results are adjusted for nationality, education level, marital status, weekly incidence of COVID-19 infections in Basel-Stadt, and hospital.  * p-value < 0.05; **p-value ≤ 0.01; *** p-value ≤ 0.001  ^§^ Score from one (poor support) to three (strong support)  CI = Confidence Interval OSSS-3 = Oslo Social Support Scale | | | |
