# Supplementary material for: Association of Different Restriction Levels With COVID-19-Related Distress and Mental Health in Somatic Inpatients: A Secondary Analysis of Swiss General Hospital Data
Source: Front Psychiatry. 2022 May 3;13:872116. doi: 10.3389/fpsyt.2022.872116 (PMC9113023; doi:10.3389/fpsyt.2022.872116)
Supplement: Supplementary file 8 [file Table_5.docx]

| **Supplementary Table 5** Changes in the mean score of distress according to the mental health assessment tools from modest to strong coronavirus disease 2019 (COVID-19) restrictions based on linear regression models (*N* = 873). | |
| --- | --- |
|  | Mean change of mental health scores (95%-CI) |
| Anxiety (GAD-7) | -0.31 (-1.14 to 0.51) |
| Depression (PHQ-8) | -0.07 (-0.95 to 0.81) |
| Somatic Symptom Disorder (SSD-12) | -1.04 (-2.79 to 0.72) |
| Mental Quality of Life (SF-36v1 MCS*) | 0.20 (-3.57 to 3.97) |
| Results are adjusted for sex, age group, nationality, education level, marital status, weekly incidence COVID-19 infections in Basel-Stadt, and hospital. *A higher score indicates better mental health  CI = Confidence Interval GAD-7 = 7-item General Anxiety Disorder questionnaire PHQ-8 = 8-item Patient Health Questionnaire SSD-12 = 12-item Somatic Symptom Disorder questionnaire SF-36v1 = Short Form 36, version 1 MCS = mental component summary | |
